# Increase in MST activity correlates with visual motion learning: A functional MRI study of perceptual learning

**DOI:** 10.1002/hbm.23832

**Published:** 2017-09-30

**Authors:** Stephanie J. Larcombe, Chris Kennard, Holly Bridge

**Affiliations:** ^1^ Wellcome Centre for Integrative Neuroimaging, FMRIB Oxford United Kingdom; ^2^ Nuffield Department of Clinical Neurosciences (NDCN) University of Oxford Oxford United Kingdom

**Keywords:** visual perceptual learning, area hMT+, functional MRI

## Abstract

Repeated practice of a specific task can improve visual performance, but the neural mechanisms underlying this improvement in performance are not yet well understood. Here we trained healthy participants on a visual motion task daily for 5 days in one visual hemifield. Before and after training, we used functional magnetic resonance imaging (fMRI) to measure the change in neural activity. We also imaged a control group of participants on two occasions who did not receive any task training. While in the MRI scanner, all participants completed the motion task in the trained and untrained visual hemifields separately. Following training, participants improved their ability to discriminate motion direction in the trained hemifield and, to a lesser extent, in the untrained hemifield. The amount of task learning correlated positively with the change in activity in the medial superior temporal (MST) area. MST is the anterior portion of the human motion complex (hMT+). MST changes were localized to the hemisphere contralateral to the region of the visual field, where perceptual training was delivered. Visual areas V2 and V3a showed an increase in activity between the first and second scan in the training group, but this was not correlated with performance. The contralateral anterior hippocampus and bilateral dorsolateral prefrontal cortex (DLPFC) and frontal pole showed changes in neural activity that also correlated with the amount of task learning. These findings emphasize the importance of MST in perceptual learning of a visual motion task. *Hum Brain Mapp 39:145–156, 2018*. © **2017 Wiley Periodicals, Inc.**

## INTRODUCTION

The human visual system is capable of improved performance following repeated practice of a visual task, a type of learning called *visual perceptual learning*. While training of most visual tasks appears to lead to location‐specific and task‐specific improvements in performance, it is also possible to design paradigms that can transfer both to other untrained visual locations [Mastropasqua et al., 2015; Xiao et al., 2008] and other tasks [Lev et al., 2014; McGovern et al., 2012; Wang et al., 2013].

Although the psychophysical literature has grown exponentially, it is only over the past few years that the neural changes underlying VPL have been investigated. Ditye et al. [2013] demonstrated that 5 days of visual perceptual learning of a motion‐color conjunction task led to changes in grey matter volume in the superior temporal sulcus (STS) that correlated with the amount of learning. Thus, even relatively short‐term training can cause structural changes in the brain. Demonstration of functional changes in neural activity also suggest that learning can modulate activity in visual areas, in particular V3a and hMT+ for visual motion tasks [Chen et al., 2016; Goldhacker et al., 2014; Shibata et al., 2016]. Interestingly, while hMT+ is strongly activated by moving stimuli, in many studies, it does not appear to show a change in response with perceptual learning, even when a motion‐related task is used [Chen et al., 2015]. The exception to this finding is the study of Goldhacker et al. [2014] who found that the change in BOLD correlated with behavioural improvement in a task requiring detection of coherent motion. The contradictory results in hMT+ response may be stimulus specific, as Thompson et al. [2013] found that some stimuli led to a reduction in hMT+ BOLD activity following training, whereas others led to an increase.

In contrast to the mixed results in hMT+, responses in area V3a are consistently altered following visual perceptual training. This has been revealed as a change in reliability of response classification in multivariate approaches in a number of studies [Chen et al., 2016; Shibata et al., 2012, 2016].

The aim of this study was to determine whether neural changes in visual areas, particularly V3a or hMT+, were associated with learning a visual direction discrimination task. Furthermore, we aimed to quantify the hemispheric specificity of any neural changes. Localizing the effects of any visual training paradigm may help the design of rehabilitation programs aiming to improve function in visual deficits such as amblyopia or hemianopia.

## METHODS

### Participants

Twenty‐eight subjects (14 female and 14 male; 19–34 years) with normal or corrected‐to‐normal vision participated in the study. All were naïve to visual psychophysical experiments. The study was approved by the local InterDivisional Research Ethics Committee (IDREC) at the University of Oxford (MSD‐IDREC‐C1–2013‐054) and all subjects gave written, informed consent. Research was carried out in accordance with the Code of Ethics of the World Medical Association (Declaration of Helsinki). Fourteen participants were assigned to the full version of the motion direction discrimination training protocol (motion training group), and the remaining 14 participants to a no‐training group (control group).

### Visual Tasks and Stimuli

Visual stimuli were programmed using Matlab (vR2012a) with Psychtoolbox (v3.0, http://psychtoolbox.org), and were presented on a CRT monitor (ViewSonic E70fSB, 1280 × 1024 pixel resolution, 75 Hz refresh rate, 17‐inch display) in a darkened room. Participants were positioned 57 cm from the screen and used a chin‐rest to minimize head movements.

**Figure 1:**
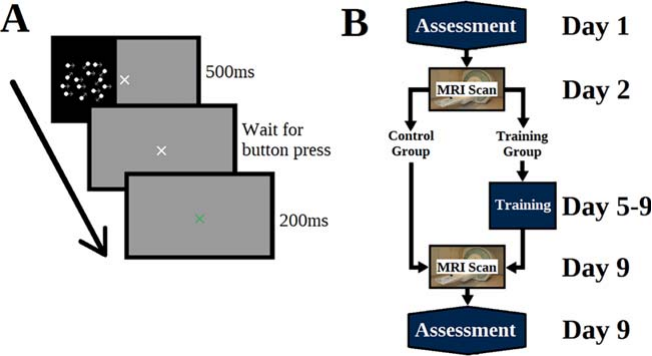
(**A**) Motion discrimination task. Participants were instructed to determine the direction of coherent motion of moving dots when presented amongst randomly moving distractor dots. Feedback was provided for correct responses (green fixation cross) and incorrect responses (red fixation cross). (**B**) Training protocol. Participation lasted for a total of 9 days. All participants completed two assessments and two MRI scans. Participants in the training group additionally completed five days of motion perception training. [Color figure can be viewed at http://wileyonlinelibrary.com]

As in a previous study [Larcombe et al., 2017], participants were asked to determine whether a group of white coherently‐moving dots (luminance = 96.8 cd/m^2^) had leftward or rightward motion, when displayed amongst randomly moving white distractor dots (“noise”; Figure 1A), presented against a black background (luminance = 0.92 cd/m^2^). The direction of coherent motion varied pseudorandomly, but was always restricted to within a 90° angle centered around the horizontal meridian. Therefore, the direction of motion presented varied within the “leftward” and “rightward” categories. Separate coherence thresholds were not calculated for these different directions of motion because the direction of motion presented varied in the same manner during the training task. Moving dots (*n* = 54) were presented within a circular area 6.6° in diameter centered 8.3° to the left or right of fixation (the edge of the stimulus aperture was 5° from fixation). The dot diameter was 0.15°, and each dot moved with a speed of 6°/s for a limited lifetime of 200 ms (12 frames), at a density of 1.5 dots/degree^2^.

### Assessment and Training Paradigms

Participation in the research study lasted for 9 days (Figure 1B). All participants in the study undertook two assessment sessions (Days 1 and 9) and two fMRI scans (Days 2 and 9). Participants in the motion‐training group completed a 5‐day protocol of motion perception training involving 10 training sessions (two sessions per day, Days 5–9). Participants in the control group completed no training and did not meet with the research team between MRI scans.

In all assessment sessions, stimuli were presented individually to the left or right visual hemifields in a pseudorandom order, with 200 trials per hemifield. For participants in the motion‐training group, stimuli during training sessions were presented in a single hemifield only. Participants were randomly allocated to train either the left or right visual hemifield (half the participants trained the left hemifield, half trained the right). Each training session (two sessions per day) consisted of 400 trials and participants were offered an optional break every 20 trials to reduce fatigue.

The difficulty of the task during both assessments and training sessions was varied adaptively following a two‐up, one‐down staircase procedure, using the implementation of Garcia‐Perez [1998]. The ratio of coherently moving dots to randomly moving dots was decreased by a factor of 0.8 following two consecutive correct responses by the participant, and was increased by a factor of 1.46 following a single incorrect response. A new staircase was initiated beginning with 80% of the dots moving coherently for each training session and for each assessment session. For the assessment sessions, two independent staircases were interleaved for the two visual hemifields.

A motion direction discrimination threshold was calculated for both assessments for each visual hemifield, and for each training session. Coherence thresholds in each session were calculated by taking the mean of the coherence on each trial in which a reversal occurred (stimulation changed from increasing in difficulty to decreasing, or vice versa). The first 10 reversal values were always discarded. This provided a discrimination threshold at which the participant is predicted statistically to be correct 80% of the time. A lower threshold (lower proportion of dots moving coherently) indicates a better performance at the task.

To quantify change in the discrimination threshold between the two assessments, a learning index was calculated using the following formula:

$$Learning\;Index=\frac{\left(T_{1}–T_{2}\right)}{\left(T_{1}+T_{2}\right)}$$

where *T*_1_ and *T*_2_ are the thresholds for the first assessment and second assessment, respectively.

### MRI Data Acquisition

All MRI data were acquired using a Siemens Verio 3 T MRI scanner with a 32‐channel head coil at the Functional Magnetic Resonance Imaging of the Brain Centre (FMRIB), University of Oxford. T1‐weighted structural brain images were acquired for each participant at 1mm isotropic resolution (MPRAGE; 192 transverse slices, TR = 2,040 ms, TE = 4.7 ms, flip angle = 8°). A gradient‐echo echo‐planar imaging (GRE‐EPI) sequence was used to acquire 312 volumes per scan run (36 transverse slices, 3 mm isotropic voxels, TR = 2,000 ms, TE = 30 ms, flip angle = 90°). Three runs of this sequence were acquired per scanner visit.

### fMRI Task Design

As for the training trials, the main task for participants in the scanner was to identify whether coherent motion was leftward or rightward. Each trial consisted of a 500 ms stimulus, 1300 ms response window, and a 200 ms feedback screen. Trials were grouped into 8‐trial blocks, each lasting 16 s.

There were 6 different stimulus types for the motion task in the scanner. These 6 stimulus blocks were as follows:

- Baseline coherence in left hemifield
- Baseline coherence +10% in left hemifield
- Noise motion in left hemifield
- Baseline coherence in right hemifield
- Baseline coherence +10% in right hemifield
- Noise motion in right hemifield.

The stimulus types were grouped in 8‐trial blocks (as described above) and participants were shown 12 blocks (2 repeats each of 6 unique stimulus types) in a pseudorandom order. After every 12 blocks, a rest block of a grey screen with white fixation cross was shown for 16s. There were 39 blocks in total, including rest blocks, during each run. There were three runs per scan session, which were later combined.

### fMRI Data Analysis

MRI data analyses were carried out using FMRIB's expert analysis tool (FEAT) v6, part of FMRIB software library (FSL; v6, http://www.fmrib.ox.ac.uk/fsl). Preprocessing of images included motion correction using MCFLIRT [Jenkinson et al., 2002] and spatial smoothing of FWHM 5 mm. Magnetic field unwarping (echo spacing = 0.56 ms, EPI TE = 30 ms, unwarp direction = −*y*, signal loss threshold = 10%), and slice timing correction (interleaved) were also applied.

T1‐weighted images were brain‐extracted using FMRIB's brain extraction tool (BET) [Smith, 2002]. Functional images were registered to T1‐weighted structural images for each participant, using FMRIB's linear image registration tool [Jenkinson et al., 2002; Jenkinson and Smith, 2001].

#### Analysis at the whole brain level

Time series statistical analysis was performed using a general linear model (GLM). For whole brain analyses, *z* (Gaussianised T/F) statistical maps of the change in BOLD activity were thresholded using clusters at *z* > 3.1 and a corrected cluster significance threshold of *P* = 0.05. Clusters were projected onto structural space for each participant, and standard space for group analysis.

A whole‐brain GLM analysis was performed for each scan run per subject. Each of the 6 block types were entered as explanatory variables (EVs) in the design matrix and linear contrasts between the EVs were computed. A higher level fixed‐effects analysis was then carried out for each subject to combine data across the three runs per scanner visit. The contrast of coherent motion compared to noise for each subject for Scan 1 and Scan 2 was extracted for the group stage analysis (no other contrasts were analyzed further).

For group analysis, images were registered to standard space, using the Montreal Neurological Institute (MNI) standard brain with 2 mm isotropic voxels, with FMRIB's nonlinear image registration tool (FNIRT) [Andersson et al., 2007]. The brain images of participants who were trained on the right visual hemifield were horizontally flipped, such that in all participants, the left visual hemifield (projecting to the right brain hemisphere) was effectively trained. This was performed so that group analysis could be carried out on participants trained on either visual hemifield. Throughout the *Results* section, the brain hemisphere labeled as “contralateral” corresponds to the brain hemisphere which is contralateral to the trained visual hemifield. For the control group, half of the brain images (chosen arbitrarily) were also horizontally flipped for consistency across groups.

Two analyses were performed on the group data. First, to determine whether changes in BOLD activity in any areas related to the amount of learning, a whole‐brain correlation analysis was carried out across all participants (both the motion training and control groups). This higher level analysis, conducted in FEAT, correlated the change in BOLD signal between scans with the amount of learning from the first to the second assessment. The regressor of interest was the learning index and regions showing a significant response showed a greater increase in neural activity with greater learning index. The second analysis calculated the change in BOLD signal between Scan 2 and Scan 1 using a paired *t* test for each of the motion training and control groups. A further higher level analysis then determined the differences between the training and control groups. Thus, any differences between the groups in the second analysis do not depend on the amount of learning.

#### Region of interest (ROI) analysis

A region of interest (ROI) analysis was carried out to calculate the percentage BOLD change in visual areas. This analysis was performed separately for Scan 1 and Scan 2 for every subject, using the mean change in activity when subjects were viewing coherent trials versus motion noise trials. The Juelich atlas [Eickhoff et al., 2005], which contains templates of visual areas based on histological data of 10 individuals, was used to create ROI masks. Masks were created of the Juelich‐defined V1, V2, V3, V4, and V5, (the latter referred to as hMT+ in this article) for each hemisphere. The full‐sized Juelich masks indicated where any of the 10 individuals used to generate the atlas showed histological evidence of the region in question [Eickhoff et al., 2005]. To minimize overlap between different visual areas, masks were thresholded at 40%, indicating that at least 6 of the 10 individuals showed histological evidence of the ROI. For hMT+, masks were thresholded at 15%, as the variability of location of this area is greater than early visual areas [Large et al., 2016]. The ROI for V3a was taken from a probabilistic atlas based on retinotopic mapping in 18 sighted control subjects [Bridge, 2011]. Within each of the visual area masks, FEATQuery was used to convert COPE values into the percentage BOLD change, for both scan meetings for each subject.

### Fixation Monitoring

An eye tracker (Eyelink1000, SR Research) was used to monitor eye fixation in all participants both inside and outside the MRI scanner. Loss of eye fixation was defined as any horizontal eye movement away from central fixation that was >2° and had a duration of >100 ms, during stimulus presentation. During assessment sessions to determine motion thresholds outside the scanner, fixation losses occurred during 6.7% of trials at the initial assessment and 3.8% of trials at the second assessment (median averages across all subjects). There was no significant difference between groups or between assessments (two‐way ANOVA with factors “group” and “assessment session”; group: *F*(1,50) = 0.07, *P* = 0.794; assessment session: *F*(1,50) = 2.0, *P* = 0.163).

## RESULTS

### The Group Trained on the Motion Task Showed Learning After Five Days of Training

The motion training group showed a significant improvement in motion direction discrimination thresholds across the 10 training sessions (two sessions per day), with the mean threshold across the group decreasing from 35.8% at Session 1 to 22.5% at Session 10.

**Figure 2:**
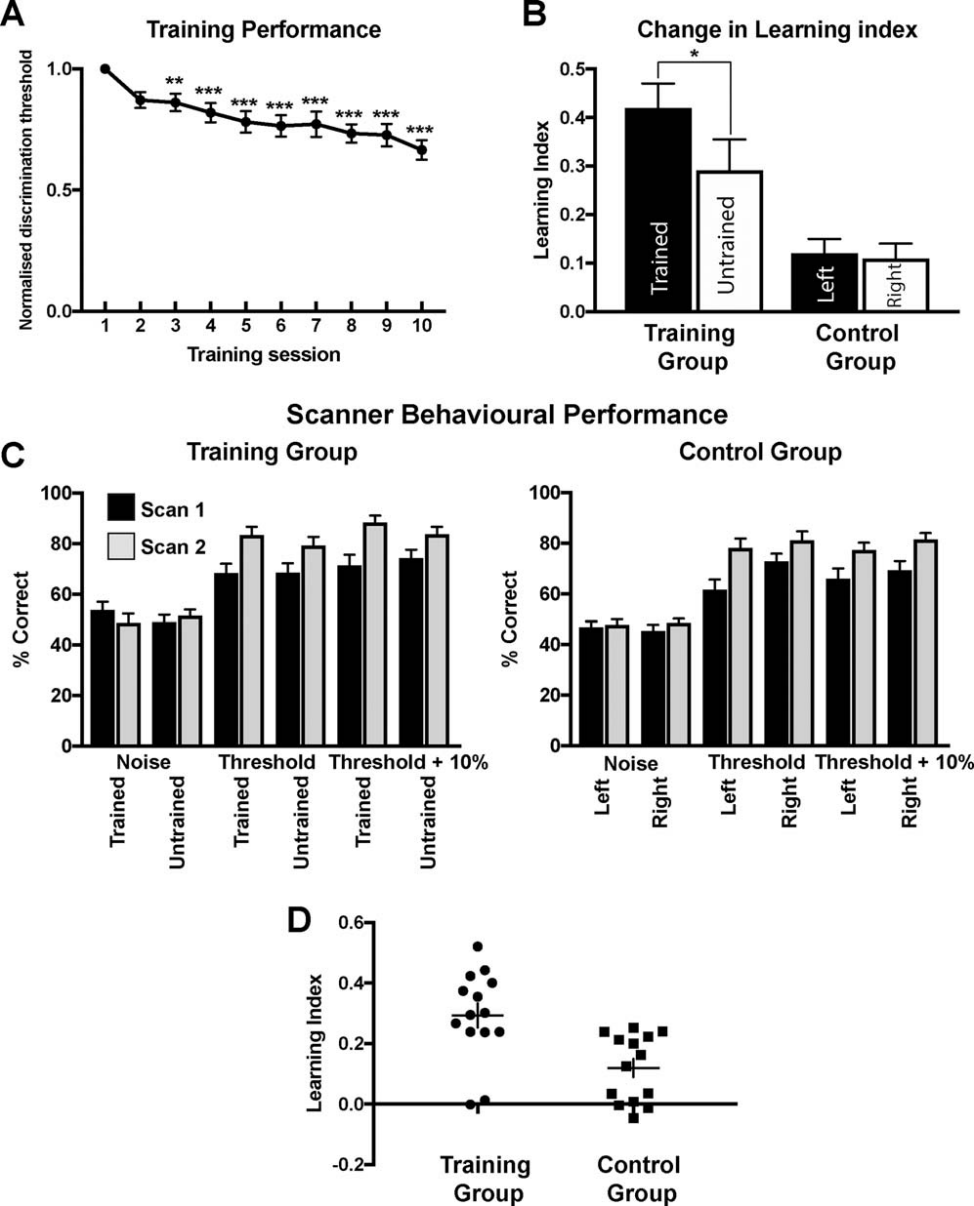
Performance of participants on the motion direction discrimination task. (**A**) The motion‐training group showed learning of the task over 10 training sessions spread evenly across 5 days of training. Motion direction discrimination thresholds gradually decreased over the training sessions, indicating a learning effect. In comparison to performance at Session 1, participants performed significantly better from Session 3 onwards. (**B**) The motion‐training group improved significantly more in the trained visual hemifield (black bar) compared to the untrained visual hemifield (white bar). There were no differences between the hemifields in the control group, where no training was completed. Furthermore, the motion training group showed significantly greater improvement on the motion direction perception task compared to the control group, reflected in the higher learning indices. (**C**) Behavioral performance inside the scanner was recorded for all participants in both groups. Participants in the training group (left graph) showed better performance than the control group (right graph), and overall performance was better in the second scan compared to the first. However, there was no significant interaction between performance in the two scans and participant group, suggesting that the trained group did not show more improvement than the control group. (**D**) The learning indices for each individual participant in the training and control groups. Note these are the same data as in B, but plotted as individual data points. *P < 0.05, **P < 0.01, ***P < 0.001. Error bars show ±SEM.

There was variability in the initial performance of the participants at the first training session, mean = 35.8% ± 17.5% (standard deviation), range = 11.6%–84.1%. Subject performance was therefore normalized across the 10 training sessions to the discrimination threshold obtained during Session 1. Analysis using nonparametric combination with a post‐hoc Fisher analysis confirmed that discrimination thresholds were lower after Session 1 for the motion trained group (*P* = 0.0002). Further analysis showed that there was significant improvement in performance from Session 3 onward, which remained significant for all other training sessions (one‐way ANOVA with unidirectional post‐hoc Dunnett, Session 1 as “control”: *F*(9,139) = 5.783; df = 9; *P* = 9.58 × 10^−7^). This learning effect is visualized in Figure 2A.

To quantify the change in performance between the pre‐ and post‐training motion perception assessments, the learning index was calculated as described above. Figure 2B shows the learning index for the trained and control groups in each hemifield separately. A two‐way ANOVA indicated the training group showed significantly greater learning than the control group (*F*(1,52) = 13.8, *P* = 0.001). There was no difference in the learning index of the hemifields across groups, (*F*(1,52) = 1.3, *P* = 0.257) nor was there a significant interaction of group and hemifield (*F*(1,52) = 2.1, *P* = 0.149).

Behavioral performance was also measured in the scanner by determining the percentage of correct responses during presentation of trials where the stimulus was at threshold, trials where the stimulus was at threshold + 10% coherence, and trials where the stimulus was noise. Each visual hemifield was assessed separately. Performance for each of these conditions is shown in Figure 2C for the trained and control groups, and quantified using a three‐way ANOVA with group, first or second scan and stimulus condition as factors. The trained group showed a significantly better performance than the control group inside the scanner (*F*(1,312) = 9.2; *P* = 0.003). Furthermore, across groups, there was a significant difference between performance at the first and second scan (*F*(1,312) = 39.0, *P* = 1.3 × 10^−9^) and according to the coherence level presented (*F*(5,312) = 72.2; *P* = 5 × 10^−50^). There was a significant interaction between performance during the first and second scan and the coherence condition presented (*F*(1,312) = 3.0, *P* = 0.002), but no interaction between any other factors (Group × Scan: *F*(1,312) = 0.006, *P* = 0.937; Group × Condition: *F*(5,312) = 0.3, *P* = 0.933; Group × Scan × Condition: *F*(1,312) = 0.5, *P* = 0.763). Thus although the trained group showed better overall performance than the control group, they did not show greater improvement between the two scans when completing the task inside the scanner. However, a direct comparison of performance in the trained and untrained hemifields of the trained group indicates significantly improved performance in the trained, compared to the untrained hemifield (paired *t* test; *t* = 2.6; df = 27; *P* = 0.016). Thus, there is some evidence for an effect of training on behavioral performance in the scanner.

### Learning Index Correlated With Change in Neural Activity of MST

**Figure 3:**
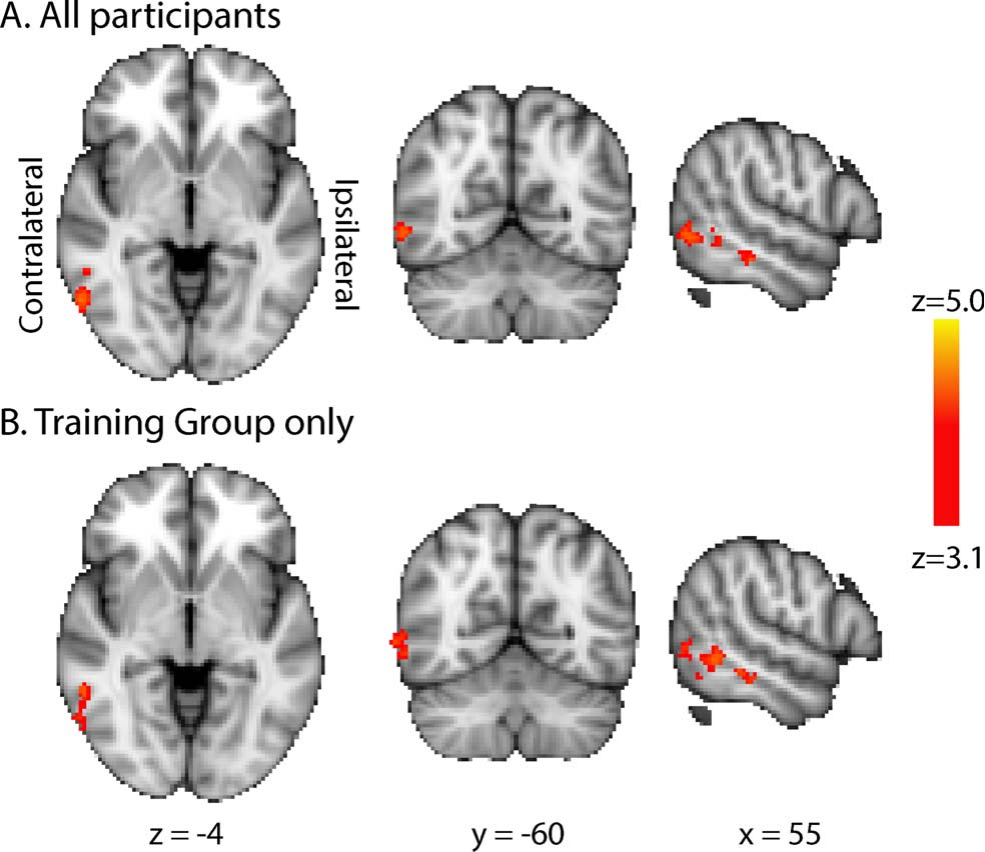
Change in BOLD signal was significantly correlated with the amount of task learning in brain region MST. (A) Whole‐brain analysis of all participants indicated that MST was the only occipital region where the increase in BOLD activity between the two scans correlated significantly with task learning. (**B**) The correlation remains significant even when only the training group is included in the analysis. [Color figure can be viewed at http://wileyonlinelibrary.com]

Given the variability in the behavioral learning index between participants even within groups (Figure 2D), this metric was used to determine the brain regions that showed a change in BOLD signal that correlated with behavior. Both groups of participants—training and controls—were included in the initial analysis. Figure 3A indicates a region of the lateral occipital cortex anterior to hMT+ that showed a significant correlation with learning. The coordinates of the center of this region (56, −62, −2, MNI space) are consistent with previous definitions of visual motion area MST [Peuskens et al., 2001]. No other regions of the occipital cortex showed an increase or decrease in response according to learning index. To ensure that the correlation was not a reflection of a difference between the trained and control groups, the analysis was repeated with just the training group included in the correlation. Figure 3B shows that the same region of MST is significant in this reduced sample.

### Beyond the Occipital Lobe There are Changes in the BOLD Signal That Correlate With Task Learning

**Figure 4:**
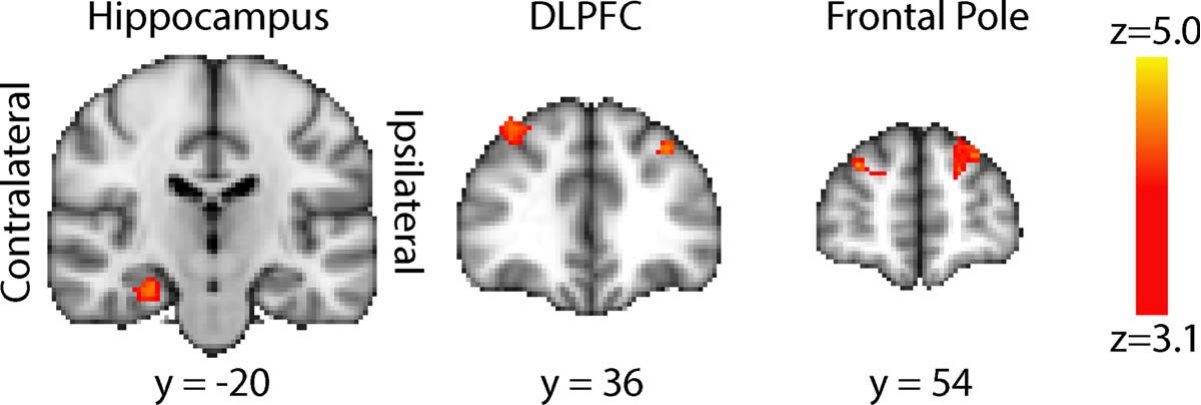
Clusters outside the occipital lobe that show a change in BOLD signal significantly correlated with the amount of task learning. The hippocampus shows a significant correlation in the hemisphere contralateral to the trained visual field. The correlations in both the DLPFC and frontal pole are bilateral. [Color figure can be viewed at http://wileyonlinelibrary.com]

The correlation analysis determined three brain regions outside the occipital cortex in which the change in BOLD signal was significantly correlated with the amount of learning across both participant groups. The anterior hippocampus showed a significant correlation in the hemisphere contralateral to the trained visual field. The center of the significantly correlated region was (28, −20, −20) and extended ventrally toward the parahippocampal gyrus. In addition, there were bilateral regions of the frontal pole showing significant correlation with peaks at (26, 58, 28) in the hemisphere contralateral to the trained visual field and (−24, 44, 38) ipsilateral. These regions are shown in Figure 4. No regions showed an inverse correlation with learning index.

### The Motion Trained Group Showed Increased BOLD Activity Lateralized to the Brain Hemisphere Contralateral to the Trained Visual Hemifield

**Figure 5:**
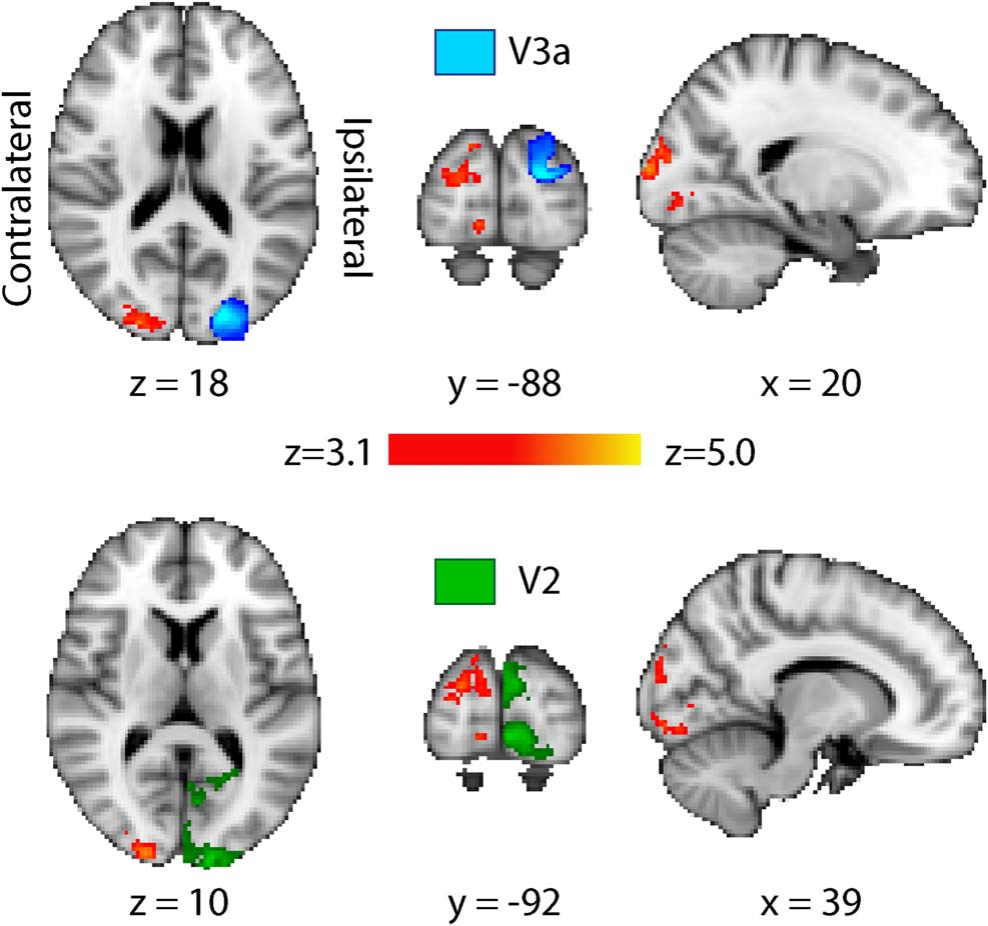
Early visual areas V2 and V3a show increased activity following training in the motion training group, compared to baseline. For the motion training group, the increase in activity is lateralized to the hemisphere contralateral to the trained visual hemifield. The red–yellow scale represents the difference in activation between the two scans. The blue region is a probabilistic map of V3a and the green is of V2, shown only in the ipsilateral hemisphere for comparison. Much of the increased activation is found in these two areas. Contralateral and ipsilateral are in reference to the trained visual hemifield. There was no significant change in activation between the two scans in the control group.

To determine whether there is an effect of the training paradigm that is not directly related to the amount of learning, the BOLD signal was compared between the two scans. Figure 5 shows the regions in the early visual cortex that show an increase in activation after training compared to before. The increase is more significant in areas corresponding to V2 and V3a only in the hemisphere contralateral to the visual field that was trained. In contrast, the control group showed no change in BOLD signal between the first and second scans. However, it is also the case that a direct contrast of the trained group compared to the control group did not show a significant difference at the threshold of *z* > 3.1, indicating that the increase in BOLD signal is not particularly robust at the current sample size and statistical threshold. No occipital regions showed a decrease in BOLD response between the two sessions in either trained or control group.

### Significant Increases in BOLD Activity are Evident in Areas V1, V2, V3, V3a, and V4 Contralateral to the Trained Visual Field

**Figure 6:**
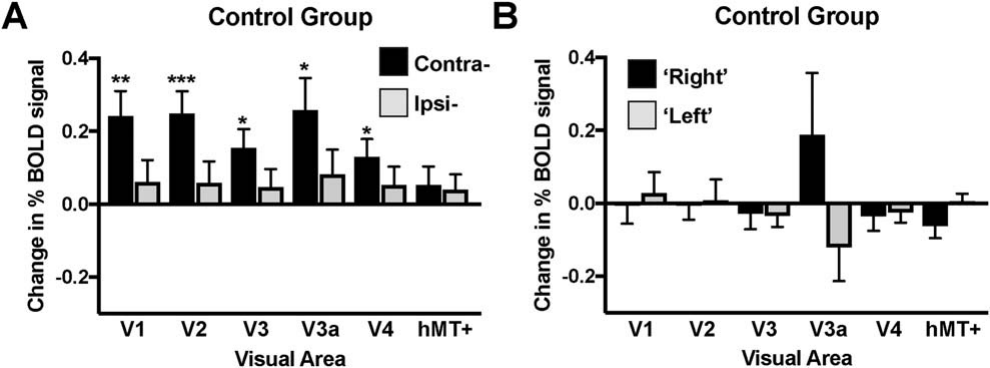
ROI analysis of the visual cortex. Five visual areas (V1, V2, V3, V4, and hMT+) were compared for each brain hemisphere. (**A**) The increase in BOLD activity in the training group at the second scan, compared to the first scan, for the hemispheres ipsilateral and contralateral to the trained visual hemifield. (**B**) The same data for the control group. Half the control data were flipped to reverse left and right hemispheres to be consistent with the training data. Thus “Left” and “Right” are assigned rather than actually reflecting those hemispheres. Asterisks indicate where one‐sample *t* test indicated change in BOLD signal was greater than zero. ***P < 0.001, **P < 0.01, *P < 0.05. Error bars show ±SEM.

The lack of a large effect of training in the whole‐brain analyses could be due to variability in the exact location of activation across participants. We therefore quantified changes in BOLD activity pre‐ and post‐training within individual visual areas V1, V2, V3, V3a, V4, and hMT+. Figure 6 shows the increase in BOLD signal between the pre‐ and post‐training sessions for training group (A) and control group (B).

A three‐way ANOVA was carried out to determine whether percent BOLD change (Scan 2 − Scan 1) differed according to group (trained or control), hemisphere (trained and untrained, right and left for control group), or visual area (V1, V2, V3, V3a, V4, and hMT+). There was a significant difference between the trained and control groups (*F*(1,312) = 18.3, *P* = 2.5 × 10^−5^), but no effect of hemisphere when compared across both groups (*F*(1,312) = 3.1, *P* = 0.081). There was, however, a significant interaction between hemisphere and group (*F*(1,312) = 9.1, *P* = 0.003). This interaction reflects the difference in BOLD signal in the trained and untrained hemispheres of the training group that can be seen in Figure 6. There was no effect of visual area (*F*(1,312) = 0.2, *P* = 0.974) and no other significant interactions between factors (Group × Visual Area: *F*(1,312) = 1.3, *P* = 0.253; Hemisphere × Visual Area: *F*(1,312) = 0.9, *P* = 0.446; Group × Hemisphere × Visual Area: *F*(1,312) = 1.9, *P* = 0.081).

## DISCUSSION

This study was designed to determine the neural effects of a five‐day visual motion training program, specifically to investigate the presence and location of plastic changes in the visual system. Previous hypotheses have suggested that improvements in performance in such visual perceptual learning tasks may be mediated by early visual areas, V3a and hMT+. The findings here are consistent with this assertion, providing further evidence for the particular role of MST, the anterior region of hMT+. This task also resulted in changes in activity of V3a and other early visual areas that were not related directly to task performance.

### Behavioral Improvement is Consistent With Previous Work

Participants who undertook the five‐day training program showed a significant improvement in their performance of the task, consistent with our previous work using the same task [Larcombe et al., 2017] and other studies, see Sasaki et al. [2010] for a review. Furthermore, in the trained group, the task improvement was mostly localized to the area of visual field that was trained over the five days. Not surprisingly the control group, who did not undertake training, showed little improvement in their task performance. During MRI scans, participants performed a direction discrimination task based on their own motion discrimination threshold from the initial behavioral assessment session. Both groups showed an improvement in performance of the task during the second scan, compared to the first scan. In the behavioral data acquired in the scanner, although the trained group showed significantly more improvement in the trained hemifield compared to the untrained hemifield, they did not show significantly greater improvement compared to the untrained group. Since performance was generally high after training, this may be due to ceiling effects and the use of a single stimulus level rather than the staircase used in the assessment to accurately quantify learning. Although we cannot rule out the possibility of context‐dependent effects affecting performance, we believe the discrepancy is more likely a function of methodological differences in determining performance.

### Elevation of BOLD Signal in MST was Correlated With Perceptual Learning of Motion Direction Perception Task

MST is the anterior portion of hMT+, and is thought to be involved in optic flow and other complex motion perception tasks [Bremmer et al., 2010; Maloney et al., 2013; Morrone et al., 2000], acting upstream of the more basic motion‐sensitive MT [Smith et al., 2006]. While MT has been shown to be equally responsive to all types of visual motion [Smith et al., 2006], MST is sensitive to global flow properties, and is most responsive to motion stimuli with flow components. As a result, MST is more responsive to coherent motion in comparison to noise‐only motion [Smith et al., 2006]. In this study, changes in BOLD signal in area MST were found to be significantly correlated with the amount of motion perception learning of the task. As the training improves the ability of participants to extract coherent motion from background noise motion, it is likely mediated by changes at the level of MST, as MT is equally responsive regardless of motion coherence. Furthermore, it has been shown that neurons in MST have motion direction selectivity [Saito et al., 1986; Van Essen et al., 1981], and activate preferentially to fields of motion [Komatsu and Wurtz, 1988], both of which were present in the motion task used here.

In this experiment, learning was targeted at the perception of coherent motion amongst noise. After training, participants who demonstrated learning required fewer coherent dots moving amongst randomly moving distractor dots (noise). As the same stimulus coherence levels were used at the first and second fMRI scans, training could have effectively made it appear as if the trials were more coherent at the second scan compared to the first scan [Liu et al., 2006].

The finding that MST is the only visual region in which the change in activation correlates with the amount of learning suggests that the change is specific to the task feature, as previously described by Shibata et al. [2016]. In their study, however, the region showing this pattern was V3a, a result not found here. This could be explained by variations in the trained stimulus. For example, here we trained a large range of motion directions delivered to a peripheral region of the visual field (5°–11.6° eccentricity). In contrast, Shibata et al. [2016] trained one specific motion direction delivered in central vision, extending to 5° eccentricity. Thus, the specific stimulus and task used for training are likely to influence the cortical region that is modified by training. Becker et al. [2008] showed that when stimulus size was large, the difference in response to coherent, compared to incoherent motion, was significantly greater in MST than the posterior region of hMT+. As suggested above, as learning the task is likely to make the motion stimuli appear more coherent, a greater correlation with learning in MST compared to hMT+ is consistent with this finding. A test for this hypothesis would be to perform a similar experiment, but training two groups with different stimulus sizes.

### Roles for V3a and hMT+ in Visual Motion Learning

While hMT+ in the human has consistently been associated with motion perception, area V3a also has a strong motion response. However, the relative contribution of these regions during and after visual perceptual learning is still under debate. Braddick et al. [2001] demonstrated that both hMT+ and V3a show activity when participants viewed 100% coherent motion compared to noise motion. This result has been confirmed by a number of other studies [Culham et al., 2001; Moutoussis et al., 2005]. Furthermore, use of continuous theta burst transcranial magnetic stimulation (cTMS) to V3a and hMT+ has also demonstrated a role in motion perception for both areas. Specifically, Cai et al. [2014] showed that stimulation of V3a disrupts discrimination of 100% coherent motion, while stimulation of hMT+ disrupts discrimination of 40% motion. They concluded that V3a computed local motion, while hMT+ computed global motion. Our study did not allow the distinction of these two motion types but, given that we found an increase in neural activity in V3a, it is possible that learning affects the computation of local motion.

More pertinent to this study is the recent demonstration that following motion training on a task with 100% coherent motion, cTMS to V3a disrupted discrimination of both 40% and 100% coherent motion, while cTMS to hMT+ no longer caused any disruption of motion discrimination [Chen et al., 2016]. This suggests that there is some transfer of the site of motion processing that occurs due to training. Here, we used the Juelich definition of V5, which corresponds to hMT+, although excludes the more anterior region that has previously been shown to correspond to human MST.

### Early Visual Areas Show a Consistent Increase in BOLD Signal

The investigation of individual visual areas showed increased activity in the early visual areas after training. This increased BOLD signal has only been found in some studies of perceptual learning, an effect that may be due to the duration of training. Jehee et al. [2012] trained participants on an orientation discrimination task over 20 days and found no change in the magnitude of the BOLD signal in visual areas V1–V4, but did find a difference in the multivariate pattern of activation. In contrast, Furmanski et al. [2004] showed a location‐specific increase in V1 activity to training on an orientation task. These findings appear to be reconciled by the study of Yotsumoto et al. [2008] who demonstrated an increase in BOLD activity in V1 at their first and second post‐training testing sessions, but the signal returned to baseline at the final testing session 4 weeks after the initial session. As the current data were acquired on the same day as the final training session, it is perhaps not surprising that we see change in these early visual areas. At a whole‐brain level, although the training group showed a significant increase in BOLD signal after training, the increase was not significantly greater than for the control group, presumably due to intersubject variability.

### All Effects Were Lateralized to the Brain Hemisphere Contralateral to the Trained Visual Hemifield

The specificity of the BOLD signal change is indicated by the lateralization to the hemisphere contralateral to the trained visual hemifield. Half the participants were trained in the left visual hemifield and half in the right visual hemifield, so this lateralization is not due to any inherent hemispheric bias, but rather the nature of the training. This type of location‐specific increase in BOLD signal has been previously identified by Furmanski et al. [2004], where the greatest change in signal in the brain corresponded to the trained region of the visual field. Here, although there was a small but significant improvement in performance on the untrained visual hemifield of the training group, there was no related increase in neural activity in the corresponding brain hemisphere. Given the small receptive fields in the early visual areas it is not surprising that any vision‐specific change in activity does not transfer at the hemispheric level. It is of interest that areas with larger receptive fields did not show increased activity between the two scans, either lateralized or bilateral. As some of these nonoccipital increases in activity were bilateral, any improvements are likely to be at an attentional or decision making level.

### Increase in BOLD Activity Correlated With Motion Perception Learning in the Hippocampus

As any type of learning requires memory, it is not surprising that motion training led to a change in activation in the hippocampus, and furthermore that the amount of learning significantly correlated with the increase in BOLD activity. Half of the participants were trained in the right visual hemifield, and half in the left. However, at the analysis stage, the data for all participants trained in the right visual hemifield were flipped horizontally, so all participants effectively trained the left visual hemifield. The change in hippocampal activity correlated with motion perception learning was lateralized contralateral to the trained visual hemifield. Although no topographic maps have been previously demonstrated in the hippocampus [Nadel, 1978], the right hippocampus receives more inputs from cortical areas in the right hemisphere, and similarly for the left hippocampus. The hippocampus also receives indirect inputs from area MST and other visual areas [Tsanov and Manahan‐Vaughan, 2008]. The data here therefore confirm the important role of the hippocampus in perceptual learning [Guggenmos et al., 2015; Manns and Squire, 2001].

### Role of Decision Making in Visual Perceptual Learning

Neurophysiological recordings during VPL have suggested that the major neuronal changes occur at the sensory decision stage rather than at a purely sensory level [Law and Gold, 2008]. Specifically, in the macaque monkey, training on a motion direction discrimination task led to a change in the responses of neurons in the lateral intraparietal region (LIP), but not motion sensitive area MT. One interpretation of these findings is that VPL leads to a change in how the task‐relevant sensory neurons are read out by higher order neurons [Kumano and Uka, 2013]. While this study also used a direction discrimination task, there was no evidence of change in neural signals in areas analogous to LIP. However, the medial prefrontal cortex (mPFC) showed increased neural activity following training and a correlation with the amount of learning across the study participants. As this region in humans seems to play an important role in both decision making and memory (reviewed in Euston et al. [2012]), it may be critical to the learning of the visual task. Furthermore, the similarity in response change to the hippocampus is also consistent with the strong link between these two regions.

## CONCLUSIONS

Here we trained participants on a visual task that involved discrimination of coherent motion direction among noise. We showed that learning of this task was correlated with changes in MST, the anterior region of hMT+. We also identified neural changes that correlated with learning in non‐visual regions of the brain, including DLPFC and hippocampus. We found changes in V2 and V3a following training, but these changes did not relate directly to the task. In conclusion, we emphasize the importance of hMT+ in visual perceptual learning of a visual motion task.
